# Altered Biomarkers of Mucosal Immunity and Reduced Vaginal Lactobacillus Concentrations in Sexually Active Female Adolescents

**DOI:** 10.1371/journal.pone.0040415

**Published:** 2012-07-10

**Authors:** Rebecca Pellett Madan, Colleen Carpenter, Tina Fiedler, Sabah Kalyoussef, Thomas C. McAndrew, Shankar Viswanathan, Mimi Kim, Marla J. Keller, David N. Fredricks, Betsy C. Herold

**Affiliations:** 1 Department of Pediatrics, Albert Einstein College of Medicine, New York, New York, United States of America; 2 Departments of Epidemiology and Population Health, Albert Einstein College of Medicine, New York, New York, United States of America; 3 Department of Medicine, Albert Einstein College of Medicine, New York, New York, United States of America; 4 Departments of Obstetrics and Gynecology and Women’s Health, Albert Einstein College of Medicine, New York, New York, United States of America; 5 Vaccine and Infectious Disease Division, Fred Hutchinson Cancer Research Center, Seattle, Washington, United States of America; University of Toronto, Canada

## Abstract

**Background:**

Genital secretions collected from adult women exhibit *in vitro* activity against herpes simplex virus (HSV) and *Escherichia coli* (*E. coli*), but prior studies have not investigated this endogenous antimicrobial activity or its mediators in adolescent females.

**Methodology/Principal Findings:**

Anti-HSV and anti-*E.coli* activity were quantified from cervicovaginal lavage (CVL) specimens collected from 20 sexually active adolescent females (15–18 years). Soluble immune mediators that may influence this activity were measured in CVL, and concentrations of *Lactobacillus jensenii* and *crispatus* were quantified by PCR from vaginal swabs. Results for adolescents were compared to those obtained from 54 healthy, premenopausal adult women. Relative to specimens collected from adults, CVL collected from adolescent subjects had significantly reduced activity against *E. coli* and diminished concentrations of protein, IgG, and IgA but significantly increased anti-HSV activity and concentrations of interleukin (IL)-1α, IL-6 and IL-1 receptor antagonist. Vaginal swabs collected from adolescent subjects had comparable concentrations of *L. crispatus* but significantly reduced concentrations of *L. jensenii*, relative to adult swabs.

**Conclusions/Significance:**

Biomarkers of genital mucosal innate immunity may differ substantially between sexually active adolescents and adult women. These findings warrant further study and may have significant implications for prevention of sexually transmitted infections in adolescent females.

## Introduction

Epidemiological studies consistently demonstrate that sexually transmitted infections (STI) disproportionately affect adolescent females [1], [2]. High-risk behaviors and lack of access to or utilization of health care services may contribute to the high prevalence of STI in adolescents. However, altered soluble mucosal immunity or lack of protective microbiota in the genital tract may also increase the risk of STI and HIV acquisition in this population. Sexual debut in the first years after menarche, when ovulation is irregular and the cervical transformation zone is highly dynamic, may render young adolescents at greater risk for STI acquisition and persistence [3]–[5]. Young women with cervical ectopy may exhibit higher concentrations of specific cervicovaginal cytokines and chemokines, which could impact susceptibility or response to genital tract pathogens [6].

Genital tract secretions contain a wide array of innate host defense molecules and represent locally-produced mediators and transudate from the systemic circulation. These molecules include human neutrophil peptides 1–3 (HNP 1-3), lactoferrin, and lysozyme, produced primarily by immune cells; IgG and IgA, secreted by plasma cells, secretory leukocyte protease inhibitor (SLPI) and β defensins, which are secreted by epithelial cells and keratinocytes. These mediators may protect against infection but, paradoxically, may also recruit and activate immune cells that serve as targets for HIV infection [7]. Vaginal bacteria also modulate host defense through multiple mechanisms [8]. Genital tract secretions collected by lavage inhibit HSV plaque formation [9], [10] and are bactericidal for *Escherichia coli* (*E. coli*) [11], [12]. This endogenous antimicrobial activity likely reflects the cumulative interactions of mediators produced by the host and commensal microbiome [13]–[15].

The extent of endogenous antimicrobial activity has not been described in genital secretions from adolescent females. We hypothesize that adolescents have altered genital mucosal host defenses compared to reproductively mature adults and conducted a cross-sectional analysis of antimicrobial activity and its potential mediators, including vaginal microbiota, in genital specimens collected from healthy, sexually active adolescent females aged 15–18 years. Outcomes for adolescents were compared to those for adult females who were participating in unrelated studies.

## Materials and Methods

### Ethics Statement

This study was approved by the Albert Einstein College of Medicine Institutional Review Board (IRB), and all clinical investigation was conducted according to the principles expressed in the Declaration of Helsinki. All participants provided written informed consent. This study involved screening for sexually transmitted infections and gynecologic care, for which minors have a legal right to confidentiality in New York State [16]. Therefore, the need for parental or guardian informed consent was waived by the IRB, and consent was not obtained from parents, guardians, or next of kin.

### Participants

Sexually active females between the ages of 15 and 18 years were recruited from the Bronx, New York, from December 2008 to May 2011 to complete a single study visit. Enrollment criteria included a reported history of vaginal intercourse. Exclusion criteria included pregnancy, breastfeeding, incident genital tract infection, or menstrual bleeding at the time of study visit; reported history of vaginal intercourse or use of douching products in the 48 hours prior to study visit; or use of oral or vaginal antimicrobial products in the seven days prior to study visit. In addition, specimens and data were collected from healthy, premenopausal adult women who were fewer than 50 years of age and who were participating in separate, IRB-approved genital mucosal immunity studies. Exclusion criteria for adult subjects included those listed above for adolescents, although some of the adult studies excluded women using hormonal contraception. Study screening procedures and specimen collection methods were identical for adolescent and adult subjects.

Urine was collected for pregnancy testing. Prior to speculum exam, vaginal pH was measured from a Dacron® swab of the lateral vaginal wall (Whatman pH paper, pH 3.8–5.5). A gynecological speculum examination was then performed. A Dacron® swab was rolled across the lateral vaginal wall and stored in 0.5 ml phosphate buffered saline (PBS) at −80°C for quantification of vaginal microbiota. Screening for *Neisseria gonorrhoeae* and *Chlamydia trachomatis* infections was performed with nucleic acid amplification testing of endocervical swabs (Gen-Probe, Inc., San Diego, CA). Subjects were evaluated for bacterial vaginosis (BV) (wet preparation with Amsel’s criteria), *Trichomonas vaginalis* (wet preparation*),* and *Candida* species (potassium hydroxide preparation). Genital secretions were evaluated for the presence of semen by an immunoassay that detects p30, a glycoprotein produced by the prostate (Abacus Diagnostics, West Hills, CA). Cervicovaginal lavage (CVL) for collection of genital tract secretions was performed with 10 ml of sterile normal saline (pH ∼5.0). Blood was collected for human immunodeficiency virus (HIV) screening by enzyme-linked immunosorbent assay (ELISA) and for serotype specific antibodies for HSV-1 and HSV-2 (HerpeSelect, Focus Diagnostics, Cypress, CA).

### CVL Samples

CVL specimens were transported to the laboratory on ice and subjected to centrifugation at 700 g for 10 minutes at 4°C. Supernatants were aliquoted and stored at −80°C.

### Quantification of Endogenous Antimicrobial Activity of CVL

The antimicrobial activity of each subject’s CVL sample against HSV-2 and *E. coli* was assessed [17]. For anti-HSV activity, Vero (monkey kidney epithelial) cells were infected with ∼50–200 plaque forming units (pfu) of HSV-2(G) mixed 1∶1 with each CVL specimen or control buffer (normal saline with 200 µg/mL bovine serum albumin). Viral plaques were counted after 48 hours. All samples were tested in duplicate in two independent experiments. For *E. coli* bactericidal activity, 3 µl of bacteria (ATCC strain 4382627) (∼10^9^ colony forming units [cfu]/ml) that had been grown overnight to stationary phase were mixed with 27 µl of each CVL specimen or control buffer (20 mmol/L potassium phosphate, 60 mmol/L sodium chloride, 0.2 mg/ml albumin, pH 4.5) and incubated at 37°C for two hours. The mixtures were then further diluted in buffer to yield 800–1000 cfu in control specimens and plated in duplicate on agar enriched with trypticase soy broth (TSB). Colonies were counted using ImageQuant TL v2005 after an overnight incubation at 37°C. All samples were tested in duplicate, with the percentage inhibition determined relative to the number of colonies on control plates.

### Quantification of Soluble Immune Mediators from CVL

The protein concentration of each CVL specimen was quantified (Micro BCA Protein Assay, Pierce Biotechnology, Rockford, IL). Interleukin (IL)-1α, IL-1β, IL-6, IL-8, interferon (IFN)-γ, IFN-α2, IL-1 receptor antagonist (ra), macrophage inflammatory protein (MIP)-1α, MIP-1β, and regulated upon activation, normal T-cell expressed and secreted (RANTES) were measured by multiplex proteome array with beads from Chemicon (Billerca, MA), quantified with Luminex^100^ (Austin, TX), and analyzed with StarStation (Applied Cytometry Systems, Sacramento, CA). Concentrations that fell below the manufacturer’s minimal detection concentration (MMDC) were set at the midpoint between zero and the MMDC.

The following immune mediators were quantified by ELISA: SLPI (R & D Systems, Minneapolis, MN), lactoferrin (Calbiochem, San Diego, CA), lysozyme (Alpco Diagnostics, Salem, NH), HNP 1–3 (HyCult Biotechnology, Uden, The Netherlands), IgG and IgA (Cygnus Technologies, Southport, NC).

### Quantification of Vaginal Microbiota Populations

Stored vaginal swabs were subjected to DNA extraction as previously described, with one sham extraction control for every 12 vaginal swabs [18]. Human 18 S rRNA gene polymerase chain reaction (PCR) was performed on all extracted DNA samples to ensure contact with vaginal mucosa during sampling and the presence of amplifiable DNA; amplification control PCR targeting a jellyfish aequorin gene was used to exclude the presence of PCR inhibitors [19]. Quantitative polymerase chain reaction (qPCR) assays utilizing primers and probes specific to each bacterium’s 16 S rRNA gene were then used to quantify concentrations of *L. crispatus* and *L. jensenii* [20]. Concentrations of *Gardnerella vaginalis*, BV-associated bacterium 1(BVAB1), BVAB2, BVAB3, and *Megasphaera* were also quantified from adolescent specimens. Four no-template controls were run with each qPCR assay to monitor for potential bacterial contamination of the PCR reagents, and (blank) extraction controls were run to assess for contamination of extraction reagents. Quantified bacterial levels were expressed as copies of bacterial DNA per vaginal swab.

### Statistical Analyses

Descriptive statistics (mean, standard deviation [SD], median, range, interquartile range [IQR]) were computed, and scatter plot graphical representations were generated. Continuous variables were compared by Student’s t test or the non-parametric equivalent. Categorical variables for each group were compared by Fisher’s exact test or by Chi squared test. Spearman’s correlation coefficients (SCC) were calculated to evaluate associations between continuous variables, and heat maps of SCC grouped by partitioning around medoids were generated. The data were clustered by an agglomerative approach, with the distance between two covariates defined as 1-SCC. Pairwise distances were calculated for all 18 variables. The first clustering was then determined by grouping together the variables with the most similar distance, with subsequent clusters determined by calculating the maximum pairwise distance within each cluster (complete linkage method).

In order to examine the relationship of *E. coli* and HSV inhibition with age, after adjusting for race, vaginal pH and HSV seropositivity, multivariable linear regression analyses were fit to the data. All tests for statistical significance were two-sided, with a p value <0.05 considered significant. Analyses were performed using SAS version 9.2 (SAS institute, Cary, NC), Graph Pad Prism Software version 5.0 (La Jolla, CA), and R version 2.12.2.

## Results

### Subjects

23 females between the ages of 15 and 18 were enrolled. Three subjects with asymptomatic chlamydia at enrollment were excluded from the analysis. Data from the remaining 20 adolescents (mean age ± standard deviation [SD] of 16.9±0.2 years) were compared to those collected from 54 adult females (mean age ± SD of 30.7±1.1 years, p<0.001) who had no evidence of incident genital tract infection (Table 1). Adolescent subjects were an average ± SD of 5±1.9 years from menarche and 2.5±1.8 years from sexual debut. Nine of the 20 (45%) of adolescent subjects reported sexual debut within two years of menarche. Nearly 30% of adolescent subjects and 14.5% of adults reported a known history of chlamydia infection (p = 0.18). Adolescent subjects were more likely to describe themselves as Hispanic (p = 0.001) and to utilize hormonal contraception (p = 0.004). At enrollment, four adolescents were using hormonal contraception (two with depot medroxyprogesterone acetate and two with tricyclic oral contraceptive pills). One adolescent subject was seropositive for HSV-2. No adolescent or adult subject reported a history of genital herpes. The mean vaginal pH in adolescent subjects was significantly higher than that in adult subjects (mean ± SD 4.8±0.08 vs. 4.5±0.07, p = 0.005), although no subjects in either group fulfilled Amsel’s criteria for bacterial vaginosis.

**Table 1:** Characteristics of study subjects.

Characteristic	Adolescents (n = 20)	Adults (n = 54)	P
Age, mean ± standard deviation (SD)	16.9±0.2	30.7±1.1	<0.001
Race/ethnicity*	0 W, 3 B, 16 H, 1 O	10 W, 24 B, 16 H, 4 O	0.001
Vaginal pH, mean ± SD	4.8±0.08	4.5±0.07	0.005
HSV seropositivity**	11 HSV-1, 1 HSV-2	36 HSV-1, 13 HSV-2	0.58, 0.1
Hormonal contraceptive use	4	0	0.004
Current cigarette smoker	1	2	1

* H = Hispanic, B = black, W = white, O = other.

** HSV serology results were available for 19 adolescent subjects.

### Endogenous Antimicrobial Activity in CVL Samples

**Figure 1:**
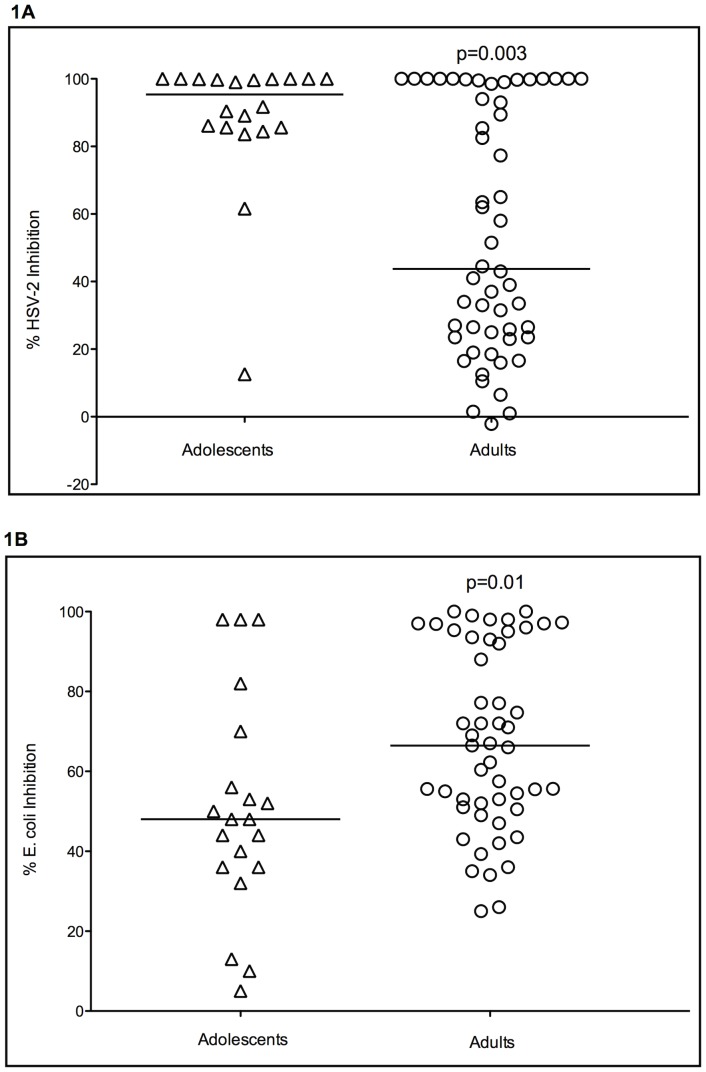
Anti-HSV (1A) and anti-*E. coli* activity (1B) for adolescent and adult cervical vaginal lavage (CVL). For anti-HSV activity, Vero cells were infected with HSV-2(G) mixed 1∶1 with each CVL specimen or control buffer. Viral plaques were counted after 48 hours and percent inhibition calculated relative to control. For *E. coli* bactericidal activity, 3 µl of stationary phase bacteria (∼10^9^ colony forming units[cfu]/ml) were mixed with 27 µl of each CVL specimen or control buffer and incubated at 37°C for two hours. The mixtures were then further diluted in buffer to yield 800–1000 cfu in control specimens and plated in duplicate on agar enriched with trypticase soy broth. Colonies were counted after an overnight incubation at 37°C and percent inhibition calculated relative to control. All samples were tested in duplicate in two independent experiments. The horizontal lines represent the median value for each group, compared by Mann Whitney U test.

CVL samples from adolescent subjects exhibited marked endogenous anti-HSV-2 activity and inhibited viral plaque formation by a median of 95.4% (range 12.6–100) (Figure 1a). CVL samples from adult subjects exhibited significantly less and more variable anti-HSV activity relative to adolescents (median percent inhibition 43.8% [range −2.1–100%]; p = 0.003). 19/20 adolescent subjects (95%) had at least 50% inhibition of HSV-2, compared to only 26/54 (48%) of adult subjects (p<0.001). Of note, CVL from one adolescent subject, who did not use hormonal contraception and who was HSV-1 seropositive, exhibited only 12.6% inhibition of HSV-2. This individual also had relatively reduced activity against *E. coli* (36% inhibition) and CVL concentrations of total protein and multiple soluble mediators that fell below the 50^th^ percentile for adolescents, including HNP 1–3, lactoferrin, lysozyme, IL-6, and IL-8.

In contrast to anti-HSV activity, endogenous anti-*E.coli* activity was significantly lower in CVL obtained from adolescent subjects compared to adults (median percent inhibition 48% [range 5–98] vs. 66.5% [range 25–100]; p = 0.01) (Figure 1b). A significantly greater proportion of adult subjects had at least 50% inhibition of *E. coli* compared to adolescent subjects (74% vs. 45%, p = 0.03).

Exclusion of the four adolescent subjects utilizing hormonal contraception did not significantly alter these results. Anti-HSV activity and anti- *E. coli* activity remained significantly higher and lower, respectively, in the remaining 15 adolescent subjects not using hormonal contraception compared to the 54 adults subjects (p = 0.01 and p = 0.04, respectively) (data not shown).

**Figure 2:**
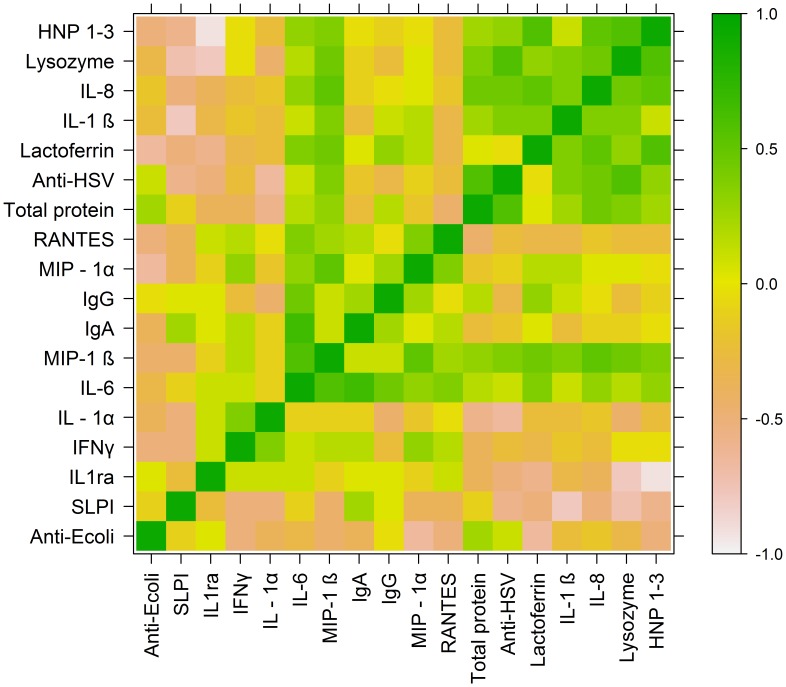
Heat map of Spearman’s correlation coefficients (SCC) for adolescent subjects. SCC were grouped by partitioning around medoids, and a color-coded heat map was generated to examine potential associations between CVL endogenous antimicrobial activity (anti-HSV, anti-*E.coli)* and concentrations of CVL soluble immune mediators in adolescent subjects.

The relationship between age and endogenous anti-*E.coli* and anti-HSV activity was further examined with multivariable linear regression models that adjusted for race, HSV seropositivity, and vaginal pH (Table 2). This analysis also identified an inverse relationship between age and anti-HSV activity, with every one-year increase in age resulting in a 0.7 percent decrease in anti-HSV activity (ß =  −0.69, p = 0.24), although this finding did not reach significance. Of note, HSV-1 and 2 seropositivity were not significant predictors of endogenous anti-HSV activity (p = 0.65 and p = 0.81, respectively), although few adolescent women were HSV-2 seropositive. In contrast, age was no longer significantly associated with *E. coli* inhibition after adjusting for the other variables in the model; only vaginal pH approached statistical significance for prediction of *E. coli* inhibition, with every 0.1 increase in pH resulting in a 1.3 percent decrease in endogenous anti-*E. coli* activity (β = −12.5, p = 0.08).

**Table 2:** Multivariable linear regression models for HSV and *E. coli* inhibition.

		HSV inhibition (N = 74)		*E. coli* inhibition (N = 74)	
		Mean ± SD 64.05±35.72		Mean ± SD 59.33±29.61	
Variable	Mean (SD) or N (%)	ß (Standard error)	p	ß (Standard error)	p
**Age**	27.51 (9.24)	−0.69 (0.59)	0.24	0.18 (0.50)	0.72
**Race**					
** White**	10 (13.51)	Reference		Reference	
** Black**	27 (36.49)	0.37 (13.74)	0.98	−4.71 (11.62)	0.69
** Hispanic**	32 (43.24)	14.12 (13.79)	0.31	−12.18 (11.66)	0.30
** Other**	5 (6.76)	21.91 (19.82)	0.27	−21.07 (16.76)	0.21
**HSV-1 seropositivity**	47 (65.28)	−0.86 (9.51)	0.93	−3.64 (8.04)	0.65
**HSV-2 seropositivity**	13 (18.06)	5.11 (13.37)	0.70	−2.70 (11.30)	0.81
**Vaginal wall pH**	4.57 (0.51)	−2.56 (8.22)	0.76	−12.54 (6.95)	0.08

### CVL Soluble Immune Mediator Concentrations

The median CVL total protein concentration was significantly reduced in adolescents compared to adults (112.5 vs. 237.5 µg/ml, p<0.001) (Table 3). We therefore compared the concentrations of specific immune mediators as absolute concentrations (unit of mediator per ml CVL) and as protein-corrected concentrations (unit of mediator per µg of CVL protein).

**Table 3:** Concentrations of cervicovaginal lavage soluble immune mediators.

Mediator	Median (Interquartile range)	Adolescents (n = 20)	Adults (n = 54)	p
Protein	µg/ml CVL	112.5 (75–239)	237.5 (149.5–386)	<0.001
HNP 1–3	pg/ml CVL	16,688 (5,067–44,093)	35,616 (11,619–246,735)	0.08
	pg/µg protein	117.5 (69.2–285.8)	76.8 (21.9–366.7	0.16
Lactoferrin	pg/ml CVL	393.5 (244.5–787.3)	474.5 (174–1,741)	0.45
	pg/µg protein	3.5 (2–5.6)	2 (0.7–6.4)	0.29
Lysozyme	pg/ml CVL	115 (73–368.3)	126 (80.9–651.3)	0.29
	pg/µg protein	1.2 (0.8–1.6)	0.8 (0.4–1.7)	0.11
SLPI	pg/ml CVL	99,843 (28,680–286,468)	237,988 (77,939–464,119)	0.03
	pg/µg protein	609.1 (243.5–1,937)	919.8 (388.8–2,012)	0.28
IgG	ng/ml CVL	1,429 (1,046–1,686)	6,684 (3,473–21,236)	<0.001
	ng/µg protein	12.5 (8.9–19.1)	33.6 (14.4–64.8)	<0.001
IgA	ng/ml CVL	365.4 (84–3–805.3)	1,104 (502.5–2,460)	<0.001
	ng/µg protein	2.4 (0.6–5.6)	4.8 (2.5–8.6)	0.04

The absolute concentrations of the six quantified antimicrobial peptides were lower in adolescents. However, the protein-corrected CVL concentrations of HNP 1–3, lysozyme, and lactoferrin were greater in adolescent subjects, although these trends did not reach statistical significance. Similarly, the protein-corrected concentrations of cytokines IL-6 and IL-1α were significantly higher in adolescent CVL specimens (p<0.001 and p = 0.005, respectively), as was the median concentration of IL-1ra (p<0.001) (Table 4). However, the ratios of combined IL-1α and IL-1β:IL-1ra were similar between adolescents and adults (0.01 vs. 0.01, respectively; p = 0.21).

**Table 4:** Concentrations of cervicovaginal lavage cytokines and chemokines.

Mediator	Median (Interquartile range)	Adolescents (n = 20)	Adults (n = 54)	p
IL-6	pg/ml CVL	5.8 (3.1–11.2)	11.4 (2.7–22.3)	0.21
	pg/µg protein	0.05 (0.02–0.08)	0.03 (0.01–0.1)	<0.001
IL-1â	pg/ml CVL	3.4 (1.8–11.8)	9.6 (1.5–2,122)	0.14
	pg/µg protein	0.03 (0.02–0.07)	0.05 (0.01–13.3)	0.6
IL-1á	pg/ml CVL	73 (28.6–146.4)	44.7 (19–97.3)	0.19
	pg/µg protein	0.6 (0.1–1.4)	0.18 (0.1–0.4)	0.005
IL-1ra	pg/ml CVL	8,857 (5,771–11,410)	7,819 (5,203–9,772)	0.39
	pg/µg protein	80.5 (31.4–111.9)	30.7 (16.6–52.5)	<0.001
IFNã	pg/ml CVL	1.04 (0.13–1.5)	1.1 (0.5–2.2)	0.61
	pg/µg protein	0.01 (0.001–0.02)	0.004 (0.002–0.01)	0.24
IL-8	pg/ml CVL	213.5 (76.9–1,408)	317.1 (97.9–1,078)	0.56
	pg/µg protein	2 (0.7–5.8)	1.6 (0.4–4)	0.38
RANTES	pg/ml CVL	2 (0.5–5.5)	2.3 (1–4.6)	0.57
	pg/µg protein	0.01 (0.01–0.04)	0.01 (0.004–0.02)	0.15
MIP-1á	pg/ml CVL	3.5 (1.8–12.4)	9.3 (4.5–18.2)	0.08
	pg/µg protein	0.04 (0.02–0.09)	0.04 (0.02–0.08)	0.75
MIP-1â	pg/ml CVL	2.9 (2.3–11.7)	7.6 (2.3–20.9)	0.06
	pg/µg protein	0.03 (0.02–0.06)	0.03 (0.02–0.09)	0.63

In contrast, IgG and IgA remained significantly lower in adolescent CVL even after protein correction (p<0.001 for IgG, p = 0.04 for IgA), with a 50% reduction of the protein-corrected concentrations observed in adults. The concentration of antimicrobial and anti-inflammatory SLPI was also lower in adolescent CVL, although the difference was no longer statistically significant after protein correction. The difference between adolescent and adult protein-corrected concentrations of CVL IL-6 was no longer significant after exclusion of the four adolescents receiving hormonal contraception (p = 0.89), but comparison of the remaining mediators was not significantly altered by the loss of these subjects.

For both adolescents and adults, anti-HSV activity correlated positively and significantly with CVL concentrations of HNP 1–3 (ρ = 0.54 adolescents, ρ = 0.31 adults), lysozyme (ρ = 0.78 adolescents, ρ = 0.42 adults), lactoferrin (ρ = 0.3 for adolescents and adults) and IL-8 (ρ = 0.63 adolescents, ρ = 0.45 adults). The anti-HSV activity also correlated positively and significantly with IL-1β, MIP-1β, and IL-6 (ρ = 0.61, 0.58, 0.39, respectively) among adolescents and with IL-1α (ρ = 0.48) among adults. Anti-HSV activity did not correlate significantly with CVL IgG in either group. In contrast to anti-HSV activity, anti-*E. coli* activity correlated positively and significantly only with protein (ρ = 0.51 adolescents, 0.6 adults). A modest correlation was noted in adolescents between anti-HSV and anti-*E. coli* activity (ρ = 0.38).

Associations between immune mediators were also analyzed by Spearman’s correlation coefficients. HNP 1–3 correlated positively with lysozyme (ρ = 0.74 adolescents, 0.7 adults), lactoferrin (ρ = 0.73 adolescents, 0.56 adults), IL-1β (ρ = 0.39 adolescents, 0.4 adults), IL-6 (ρ = 0.52 adolescents, 0.54 adults), IL-8 (ρ = 0.73 adolescents, 0.68 adults), MIP-1β (ρ = 0.58 adolescents, 0.55 adults) and protein (ρ = 0.47 adolescents, 0.5 adults).

**Figure 3:**
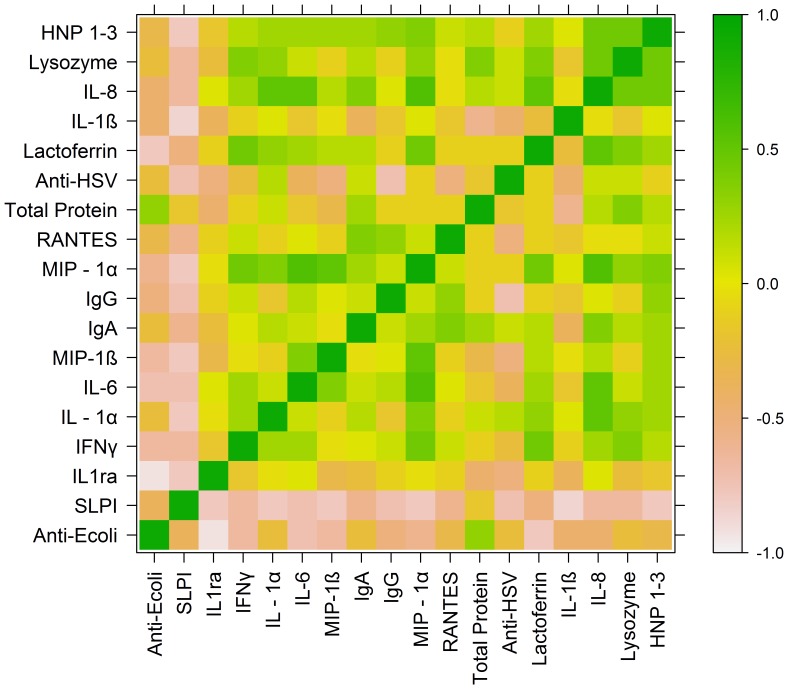
Heat map of Spearman’s correlation coefficients (SCC) for adult subjects. SCC were grouped by partitioning around medoids, and a color-coded heat map was generated to examine potential associations between CVL endogenous antimicrobial activity (anti-HSV, anti-*E.coli)* and concentrations of CVL soluble immune mediators in adult subjects.

Heat maps were generated to represent correlation coefficients and the degree of relatedness between anti- *E.coli* activity, anti- HSV activity, and soluble immune mediators for adolescents (Figure 2) and adults (Figure 3). Heat map analysis for adolescents identified a block of high correlation (indicated by green coloration) consisting of anti-HSV activity, HNP 1–3, lysozyme, lactoferrin, IL-1β, IL-8, and total protein. This structural relationship was not observed in the heat map analysis for adult subjects, suggesting that these variables were not as tightly correlated with one another in adults. For adolescents, a second cluster comprised of IL-6, MIP-1β, IgA, IgG, MIP-1α, and RANTES indicated a strong degree of relatedness between these variables. The association between these variables was more modest in adults.

### Vaginal Swab Bacteria Concentrations by qPCR

The modest but significant increase in vaginal pH observed in adolescent subjects, combined with the lower levels of endogenous activity against *E. coli* in this group, suggested the possibility of altered vaginal lactobacillus populations in adolescent subjects relative to adult women. Recent studies suggest that lactobacillus proteins contribute to the anti-*E. coli* activity in CVL (Kalyoussef, *et al*, submitted). We therefore quantified concentrations of *Lactobacillus jensenii* and *crispatus* for all 20 adolescents and for the 24 adult subjects that had vaginal swab specimens available (Table 5), as these two species have been associated with a healthy vaginal environment [21]. In addition to the difference in age (mean age ± SD 31.1±8.7 in adults vs. 17±0.9 in adolescents, p<0.001), there were also differences in race/ethnicity. Specifically, 38% of adults were Hispanic and 54% were black; 80% of adolescents were Hispanic and 15% were black (p = 0.03) (the remaining subjects were white or of another racial/ethnic category). Although the proportions of adolescent and adult subjects with detectable concentrations of *L. jensenii* were similar (57% vs. 58%, p = 0.36), the median level of *L. jensenii* DNA observed in the 20 adolescent subjects was significantly reduced relative to adults and was close to the lower limit of detection (62.5 DNA copies/swab in adolescents vs. 3.4×10^4^ DNA copies/swab in adults, p = 0.004). The proportions of subjects in each group with detectable levels of *L. crispatus* were similar (p = 0.77), with relatively low and similar concentrations in adolescents and adults (4.2×10^3^ DNA copies/swab vs. 2.8×10^4^, respectively; p = 0.31). A positive but modest correlation was noted between *L. crispatus* concentration and anti-*E. coli* activity when adolescent and adult data were combined (ρ = 0.3).

**Table 5:** Concentrations of lactobacillus bacteria in vaginal swabs.

	Adolescents	Adults	p	Adolescents	Adults	p
	(n = 20)	(n = 24)		(n = 20)	(n = 24)	
	No. PCR+	No. PCR+		DNA copies/swab, median	DNA copies/swab, median	
*L. crispatus*	12	13	0.77	4.2×10^3^	2.8×10^4^	0.31
*L. jensenii*	8	14	0.36	62.5	3.4×10^4^	0.004

In addition, extracted DNA from vaginal swabs collected from adolescent subjects was available for quantification of *Gardnerella vaginalis*, *Megasphaera*, and Clostridia-like BVAB1, BVAB2, and BVAB3. These organisms have been shown in prior studies of adult women to be sensitive (*G. vaginalis*) and specific (*Megasphaera*, BVAB1–3) indicators of bacterial vaginosis [22]. All adolescent subjects had detectable concentrations of *G. vaginalis* DNA recovered from vaginal swabs, with a median of 5×10^6^ DNA copies/swab (range 1.6×10^4^−3.3−10^10^). 7/20 (35%), 6/20 (30%), 9/20 (45%), and 7/20 (35%) of adolescent subjects had detectable concentrations of *Megasphaera*, BVAB1, BVAB2, and BVAB3, respectively. The median concentration of *Megasphaera* recovered from adolescent vaginal swabs was 300 DNA copies/swab (range 300−1.4×10^8^). The median concentrations of BVAB1, 2, and 3 were 1.6×10^3^ (range 1.6×10^3^−1.8×10^9^), 25 (25−1.4×10^7^), and 90 (90−6.2×10^7^), respectively.

## Discussion

To our knowledge, this is the first study to examine genital secretion antimicrobial activity and its potential mediators in adolescent females. We found that CVL collected from a cohort of sexually active adolescents had significantly more endogenous anti-HSV activity relative to older women. This finding correlated with higher concentrations of specific cytokines and antimicrobial proteins (lactoferrin, lysozyme and HNP 1–3) in adolescent CVL, although for some cytokines and chemokines, the CVL concentrations were low and the differences between groups modest, thus the physiological impact of any differences noted is unclear. In contrast, adolescents exhibited lower CVL levels of SLPI, IgG, and IgA, reduced CVL anti-*E. coli* activity, and lower concentrations of vaginal *L. jensenii*.

A multivariable linear regression model identified a non-significant association between increasing anti-HSV inhibition and decreasing age, which could suggest that age was a surrogate marker for a more significant variable not assessed in our study, such as cervical ectopy or immune cell populations. Chemokine, cytokine, and antimicrobial peptide secretion may differ substantially between ectocervical, endocervical, and uterine microenvironments. For example, endocervical and uterine cell lines secrete significant levels of the lymphocyte and macrophage inflammatory protein 3 alpha (MIP-3α) in response to inflammatory stimuli [23]. Thus the presence of cervical ectopy and increased proportion of columnar epithelium could contribute to differences in soluble mediators and endogenous antimicrobial activity. Age may also be an indirect marker of genital mucosal changes associated with onset of sexual activity; in an older cohort of sexually active 15–24 year old women, we identified a significant and inverse relationship between endogenous anti-HSV activity and years from sexual debut, independent of age (Madan *et al*, unpublished data). In addition, adolescents in our study were more likely to utilize hormonal contraception. Although excluding the four subjects utilizing hormonal contraception did not change our results, the potential impact of this variable requires further investigation in larger studies of adolescents.

Importantly, the results of this study suggest several potential mechanisms that could contribute to genital mucosal immune activation and an increased risk of HIV and STI acquisition and transmission among adolescents. For adolescents, SCC and heat map analyses identified a tight, “clustering” association between anti-HSV activity, HNP 1–3, lysozyme, lactoferrin, IL-1β, and IL-8. An increase in antimicrobial factors such as HNP 1–3, lysozyme, and lactoferrin, released by degranulating neutrophils recruited in response to IL-8 [24], may contribute to endogenous genital secretion activity against HIV and HSV [25]–[30]. However, at least one study that correlated genital secretion HNP 1–3 levels with *in vitro* activity against HIV also identified an independent association between increased HNP 1–3 levels and HIV acquisition risk [7]. The authors hypothesized that increased HNP 1–3 levels may have led to an influx of HIV target cells, thus increasing the risk of HIV acquisition. The lower levels of anti-inflammatory and antimicrobial SLPI we observed in adolescents may also contribute to a net increased inflammatory milieu; lower concentrations of vaginal SLPI have been associated with increased risk of HIV acquisition [31]. This paradigm of genital mucosal immune activation is consistent with a recent study correlating higher genital tract inflammatory cytokines in the setting of acute HIV infection with higher viral load set points and greater CD4 depletion [32], [33].

The lower overall protein levels in adolescent CVL merit further study, as the specific proteins measured represent <2% of the total protein. IgG and IgA were significantly lower in adolescents, both as absolute CVL and protein-corrected concentrations. A prior study of three adolescent females identified steeper peri-ovulatory drops in cervical IgG relative to the nadir observed in sexually mature women [34]. We collected a single specimen from each subject and did not account for stage of menstrual cycle, but the consistently low IgG and IgA concentrations we observed, along with the narrow IQR for these mediators (IgG 1,046–1,686 ng/ml; IgA 84.3–805.3 ng/ml) suggest a reduction of immune globulin not wholly attributed to menstrual cycle variability. Diminished cervicovaginal IgA could contribute to the high prevalence and recurrence rate of chlamydia observed in adolescents [35]–[39].

In contrast to the increased anti-HSV activity, we observed lower endogenous anti-*E. coli* activity in adolescent CVL specimens. Anti- *E. coli* activity correlated with anti- HSV activity in adolescents but not in adults, suggesting the possibility that mucosal factors may differentially contribute to endogenous antimicrobial activity in these two groups. However results of multivariable analysis suggest that the reduced anti-*E.coli* activity observed in adolescents is less related to age and more related to the finding of increased vaginal pH in this group. Thus reduced anti-*E.coli* activity may reflect paucity of *L. jensenii* and not a loss of human-derived CVL antimicrobial peptides [12]. Only 40% of adolescents in our study had detectable *L. jensenii*, and the median adolescent *L. jensenii* concentration was four log units below that observed in adults (p = 0.004). The combination of diminished *L. jensenii* and prevalence of bacterial vaginosis-associated organisms (Megasphaera, BVAB1–3) [18], [22], [40] in adolescent subjects suggests a high prevalence of asymptomatic BV and may contribute to the reduced concentration of SPLI observed in this group [41]. BV represents a significant risk factor for HIV acquisition [42], possibly by increasing concentrations of activated endocervical CD4+ T cells [43], and has been associated with increased genital tract HIV-1 shedding [44], thereby potentiating the risk of transmission.

Larger studies with rigorous adolescent-to-adult matching for race, ethnicity, and hormonal contraceptive use are needed to address the limitations of this study. These studies should include sampling throughout a menstrual cycle, cervical cytobrush evaluation of immune cell populations, more complete analyses of vaginal microbiota, and assessment of intercurrent HPV and HSV shedding. Prospective studies are necessary to delineate the potential relationship between endogenous antimicrobial activity and risk for STI acquisition.

The results of this cross-sectional analysis, however, suggest several key differences between the adolescent and adult genital tract environment. We speculate that these differences may translate to an increased *in vivo* susceptibility to HIV and other STI and portend the need for unique methods of STI and HIV prevention in adolescent females.
